# Burden, Positive Aspects, and Predictive Variables of Caregiving: A Study of Caregivers of Patients with Pediatric Glaucoma

**DOI:** 10.1155/2019/6980208

**Published:** 2019-08-26

**Authors:** Yingting Zhu, Jinyun Gao, Xinyan Li, Qiongman Yang, Yu Lian, Huiming Xiao, Wenmin Huang

**Affiliations:** State Key Laboratory of Ophthalmology, Zhongshan Ophthalmic Center, Sun Yat-sen University, Guangzhou, Guangdong, China

## Abstract

**Purpose:**

To determine the presence of burden and positive aspects among caregivers of patients with pediatric glaucoma (PG) and to establish whether they were influenced by the characteristics of the caregivers and the patients.

**Method:**

This study was designed to be cross-sectional and descriptive. The Chinese version of the Caregiver Burden Inventory (CBI) and the Positive Aspects of Caregiving (PAC) questionnaires were used to evaluate the caregivers. The demographic data of the enrolled participants were also collected. The CBI and the PAC scores were analyzed in relation to each other and different characteristics of the patients with PG and their caregivers through a generalized linear regression model.

**Result:**

Most of enrolled 57 caregivers were found to act with a moderate level of burden and benefits. The emotional subscale score of the CBI is negatively related to the aggregate score of the PAC and to that of outlook on life. Moreover, patients with a longer duration of disease and caregivers who were female, had jobs, had lower education levels, and had lower household incomes have qualities that are positively related to the summary score of the CBI. However, no factors we collected were significantly related to the PAC score.

**Conclusion:**

The results suggest that caregivers perceive positive and negative experiences in the care and support of patients suffering from PG. In addition, we should pay more attention to the caregivers with a high risk of experiencing caregiver burden, especially females with jobs and also with lower incomes and lower levels of education.

## 1. Introduction

Glaucoma accounts for approximately 2% to 15% of the cases of blindness in infancy and childhood all over the world [1]. Pediatric glaucoma (PG) shows different rates of reported incidence in different areas, and these rates are especially higher in low- and middle-income countries [2, 3]. PG, both primary and secondary glaucoma, is a devastating vision-threatening condition characterized by lifelong therapy and periodic follow-up, which brings great challenges and burden for children and their families, especially their primary caregivers [4].

During the provision of delicate care for the children with illness, tremendous sacrifice and stress will lie with the caregivers. The stress and responsibility adversely affecting the caregivers are defined as caregiver burden. Caregiver burden has been reported in many chronic pediatric diseases, such as children with allergies [5], asthma [6], obesity [7], cerebral palsy [8], and epilepsy [9]. It has been reported that children with primary congenital glaucoma and surgery will significantly affect their caregiver's quality of life (QoL) [10, 11], their emotional burden, and even their severity of depression [4]. However, insufficient attention has been paid to the caregiver burden in Chinese parents of children with PG. Moreover, positive experiences of caregiving also lack sufficient evaluation. Caregiving can also contribute to an improvement in satisfaction and even in QoL, which has been identified in caregivers of individuals with dementia [12, 13] and brain injury [14]. However, there is a paucity of studies of PAC in caregivers of patients with PG. Therefore, we initiated this study to evaluate the Chinese caregiver's burden and positive experiences, as well as the association between them. Meanwhile, we aim to investigate the subjective factors affecting caregivers' negative and positive aspects to provide new insights into the more comprehensive acknowledgment of caregiving behaviors and experiences.

## 2. Methods

In the present study, PG patients and their primary caregivers were enrolled at Zhongshan Ophthalmic Center, Sun Yat-sen University, from July 2016 to July 2017. The study conformed to the tenets of the 1995 Declaration of Helsinki, and informed consent was obtained from the enrolled subjects.

The demographic data of the enrolled patients (i.e., age, gender, times of operation, eyes, and duration of disease) and their caregivers (i.e., age, gender, occupation status, education level, household income) were collected.

PG was defined, according to the criteria from the British infantile and childhood glaucoma (BIG) eye studies [15], as (1) intraocular pressure (IOP) >21 mmHg, (2) large disc cupping (>0.3) or disc asymmetry, (3) enlarged corneal diameter, corneal edema, or Descement's membrane splits, (4) progressive myopia or enlarged axial length (with growth rate greater than normal), and (5) visual field defects. The diagnosis of PG should meet at least two or more of the above criteria. The caregivers were parents of the children with PG.

Overall, caregivers were evaluated using the Chinese version of the Caregiver Burden Inventory (CBI) [16] and the Positive Aspects of Caregiving (PAC) questionnaire [17]. The CBI assesses multidimensional aspects of the caregiver burden, including physical (questions 1 to 4), emotional (questions 6, 8, 12, 13, and 14), social (questions 5, 7, 9, 10, and 11), time-dependence (questions 15 to 19), and developmental (questions 20 to 24) burden. Each aspect was scored from 0 to 4, with higher scores indicating higher burden levels. The aggregated values range from 0 to 96, with a score between 0 and 32 graded as mild burden, between 33 and 64 graded as moderate burden, and between 65 and 96 graded as severe burden.

The PAC assesses two aspects, including self-affirmation (questions 1 to 6) and outlook on life (questions 7 to 9). There are 9 items, and each aspect is scored from 1 to 5 (1 = *disagree a lot*, 2 = *disagree a little*, 3 = *neither agree nor disagree*, 4 = *agree a little*, and 5 = *agree a lot*) and scored 0 if the caregivers reply “unknown” or “refuse to answer.” The sum of the scores was calculated to measure the levels of burden or positive aspects of caregivers. The aggregated value ranges from 0 to 45, with a score between 0 and 15 graded as low, 16 to 30 graded as moderate, and 30 to 45 graded as high.

## 3. Statistical Analyses

Statistical analyses were conducted using SPSS software (version 20, SPSS, Inc., Chicago, Illinois, USA). Continuous variables are presented as the mean ± standard deviation/median (min–max). An analysis of the relationship between the aggregated and each aspect value of the CBI, the PAC, and related factors (demographic data for the enrolled patients and caregivers) was conducted using a generalized linear regression model. The level of significance was set at *P* < 0.05.

## 4. Results

In the present study, we enrolled 57 patients and their respective caregivers. Their demographic data are shown in Tables 1 and 2. The scale, average, and range of scores on the CBI and PAC are shown in Tables 3 and 4. The magnitude of the CBI scores of most enrolled subjects was graded as mild (23, 40.4%) or moderate in terms of burden (29, 47.4%), while only 5 subjects scored higher than 65 and were considered a severe burden. Regarding the PAC scores, thirty-one of the subjects were categorized as moderate, 9 subjects were categorized as mild, and 17 subjects were categorized as severe. Among the five aspects of the CBI, the order of the average score for each item is as follows: time-dependence (2.54), physical (1.88), development (1.87), social (1.17), and psychological (0.60). Among the 24 questions, the top five items with highest scores are “I have to watch my care-receiver constantly” (Question 17, 3.22), “I expected that things would be different at this point in my life” (Question 24, 2.93), “I have to help my care-receiver with many basic functions” (Question 18, 2.92), “I'm not getting enough sleep” (Question 1, 2.69), and “My care-receiver is dependent on me” (Question 16, 2.66). Among the two aspects of the PAC, the average score for self-affirmation was higher than that for outlook on life. Among the 9 questions, the top two items with highest scores are “It has enabled you to develop a more positive attitude toward life” (Question 8, 3.57) and “It makes you feel needed” (Question 3, 3.07).

The relationship between the CBI score and the PAC is shown in Table 5. Only the psychological score of the CBI was negatively related to the aggregate score of the PAC and the score for outlook on life. The generalized linear regression analysis of the demographic factors of the CBI score is summarized in Table 6. CBI scores differed by the duration of the disease, gender, occupational status, education level, and household income of caregivers but not by the other factors. Compared with patients whose duration of disease is more than 1 year, patients with a short duration of disease bring less burden to their caregivers. Moreover, compared with caregivers who are male, unemployed, less educated, and with less household income, caregivers who are female, working, and more educated and have more household income suffer a greater burden. However, we did not find any correlation between the demographic factors and the summary scores of the PAC.

As shown by more detailed analysis, caregivers with lower education levels suffer a greater burden from aspects of development. Being female and working are positively related to the physiological burden. Patients who are younger and have undergone a longer disease duration and caregivers older in age and lower in household income show positive associations with the time-dependent burden (Tables 7–9).

## 5. Discussion

PG is the leading blinding condition in infancy and childhood. Most of these patients are in need of surgical intervention, with 31.6% of our study sample requiring two or more operations, which is similar to the findings of other studies [13, 18]. Even with proper management, the progression of PG is notoriously difficult to control due to late and challenging diagnosis. Vision-threatening progression requires comprehensive and long-term follow-up, which will affect the QoL of the patients affected by PG, as well as that of their caregivers [9, 10].

The caregiver burden was initially studied and conceptualized in the 1960s [19–21]. The most common concepts include physiological, psychological, emotional, social, and financial burden, with each representing different aspects of the stress and depression of caregivers [11, 22, 23]. The deterioration of both the physical and psychological health of caregivers was likely to result from caregiver burden, which also led to negative feedback directed toward the care recipients [24, 25]. In the present study, the caregivers of PG patients were found to act with a mild or moderate level of burden, as shown by the CBI questionnaire, which is less serious than the findings of other studies using another assessment instrument called the Caregiver Burden Questionnaire [10]. However, there have been few reports of positive experiences of caregivers of children with PG. PAC was reported as appraisal, satisfaction, sense of accomplishment, improvement of family relationship, and meaningful life expectations [26, 27]. It was widely illustrated that negative and positive experiences are both essential to the health of caregivers and the healthcare received by care recipients [12, 28]; therefore, we emphasize both to render a better understanding of overall caregiver experience. Our study demonstrated that despite mild or moderate caregiver burden, caregivers of PG patients present a moderate level of positive aspects. Identifying the predictive factors of caregiver burden and its positive aspects could improve our cognition and strategy of preventing and treating this type of illness. Consequently, the association between the CBI and PAC arouses our interest, as well as their potential predictors.

Based on the results of the generalized linear regression analysis, our study found that the aggregate score of the CBI was related to several demographic factors of the patients and caregivers themselves. First, the length of disease duration is positively associated with the aggregate score of the CBI. A long course of the disease could be the consequence of late presentation, continuous follow-up, multiple surgeries, or even worse, visual acuity, which all apparently exert challenging impacts on the caregivers. Second, compared with caregivers with a bachelor's degree or above, caregivers with an education background of primary school or below experience greater burden. Another study reported that caregiver education levels were associated with children's dental caries. As the study shows, if the caregivers are less educated, they may have more difficulty in understanding the disease, and it could take more time for them to learn how to take care of the patients [29]. Further studies should be designed to examine the relationship between caregivers' education level and their behavior, such as compliance with the doctors' advice, increased knowledge about PG and other health-related topics, frequency of follow-up, or even their lifestyle. In the present study, two-thirds of the enrolled caregivers are female, half of them are parents with jobs, and more than 80% are caregivers with low household income, which are all significantly related to the caregiver burden. Given that patients with PG are mostly infants and children, it is not surprising that mothers play a more essential role in the daily caregiving of the children than fathers do. Nevertheless, the caregiving stress of the mother especially contributes to the negative effects on the QoL of children with chronic disease [30, 31]. However, we also do not exclude fathers to avoid omitting their voices as partakers in caregiving roles. In addition, despite half of the caregivers being employed, most of their families have low incomes. Due to the chronic disease process and the repeated follow-up requirement, poor families are more vulnerable to bear greater financial difficulties. Prior literature has reported that underprivileged parents were at high risk of having depression and anxiety during child rearing, especially those who lived below the national poverty level [32]. Moreover, several studies have documented that depressed caregivers were more likely to suffer from financial poverty, unemployment, and divorce, which would exacerbate the low quality of healthcare they are able to provide [33, 34].

As our detailed analysis shows, among the five aspects of the CBI, the time-dependence burden shows the highest average score. The time-dependence subscale investigates the time cost of caregivers. Three of five items from this subscale are listed in the top five burdens of the entire CBI assessment. The young ages of the PG patients, long course of disease, low household income, and young age of caregivers are all significantly related to the time-dependent burden. Obviously, younger patients are dependent on their parents, which will occupy a large amount of their caregivers' time. The natural chronic process of PG requires extended and meticulous nursing and caring, which could bring heavy time pressure on the caregivers. In relation to the age of caregivers, there has been much division among different literature sources. Some observers have found that older caregivers suffered from a greater burden as a result of longtime interactions with the patients and the chronic course of the disease, similar to dementia [35] and schizophrenia [36]. Other observers indicate a reverse relationship between burden and age in caregivers of patients with mental illness [37]. Caregivers of younger children usually lack experience with parental caring, let alone caring for infants or children with illnesses.

The second-highest score is from the developmental burden, which assesses caregivers' feelings of impediment to their development compared to those of their peers [38]. Decreased personal time limits the progress of career, social life, and even hobbies to some extent. Caregivers with lower educational levels are prone to be affected.

The third-highest score is that of the physical aspects, which describes the feeling of fatigue and damage to the physical health of the caregivers [38]. The physical burden presents as a shortage of self-care and the occurrence of somatic symptoms, such as headache, insomnia, and a decline in immunological function. Caring for babies and children usually deprives parents of substantial amounts of sleep, so sleeplessness exerts damage to the physical health of the caregivers. In our present study, the gender and working status of caregivers are both related to their physical burden. This indicates that professional female caregivers were exposed to both physical and mental stress.

Among the 9 items of PAC, the top two highest items are “Enabled you to develop a more positive attitude toward life” from aspects of life expectation and “It makes you feel needed” from those of self-affirmation. It thus appears that when caregivers provide attentive care to their PG children, it could also bring positive feedback to the caregivers themselves, especially the feeling of being needed, which could strengthen their sense of satisfaction and gain. Most of the subjects experienced moderate or high levels of positive feelings during caregiving, which indicated a still-optimistic situation for PG caregivers.

As our present study showed, aggregate scores of the PAC and “outlook on life” subscales were both negatively associated with emotional burden. The emotional burden subscale had the lowest scores in our study. The emotional burden describes the negative feelings of the care receivers due to their unpredictable and bizarre behaviors [15]. This finding suggests that the behavior of PG patients is critical to the positive aspects of caregiving. Obviously, the behavior of PG patients is the most direct reflection of parental caring in family daily life. Some PG patients must use eye drops everyday with or without the help of their caregivers, even more than once. Moreover, due to the poor vision of PG patients, they are unable to perform common sports activities that other healthy children can perform, even attending normal primary school if they are blind. They could bring a feeling of uniqueness, embarrassment, and discomfort to the PG patients as well as their caregivers.

Social burden describes the role conflicts, especially in the family relationships of the caregivers. In relation to other family members or relatives, caring for children with illnesses merits cultural valuation and support from other family members could reverse the depression and strain from caregiving, which indicates that family support and understanding are vital to caregivers' optimistic expectations of life [38]. Regarding the relationship between caregivers and care recipients, it was reported that the intensity of anxiety and depression was related to the chronicity and severity of disease, which was further related to the quality of life or even the personality of PG children. Therefore, the natural course of the disease and the daily behaviors of PG children will affect the affinity between caregivers and their care recipients [9, 10]. As our analysis showed, caring for PG patients contributes to a relatively low level of social burden. However, no associated predictor was found.

With regard to the research methods, some limitations need to be acknowledged. First, a large sample size is needed to enhance our power to evaluate the predictive factors of caregiver burden and the positive aspects associated with caregiving. In addition, long-term follow-up should be reinforced in future research to detect changes in the caregiving experience. Furthermore, additional dimensions of predictive variables should be evaluated for greater understanding of the experience of caring for PG patients, such as sleep quality, medical compliance, and awareness of disease.

In conclusion, our study indicated that the caregivers of PG patients suffer from burden and stress, especially in time-dependence, physical and development aspects. However, to a certain extent, positive aspects were also found among the caregivers, which serve as optimistic feedback of their psychological status. Therefore, as doctors of PG patients, we should provide appropriate guidance and psychosocial intervention to the caregivers, which is beneficial for the physical and mental health of both the caregivers and the children receiving their care. In addition, we should pay more attention to distinctively vulnerable groups of caregivers, such as employed females with low incomes and low levels of education. Specific strategies should be considered and implemented to prevent or mitigate caregiver burden and to enhance and encourage caregivers' positive experiences.

## Figures and Tables

**Table 1:** Demographic data of the enrolled patients.

Variable	*n* (%)	Mean (SD)
Age (in months)		30.09 (35.11)
Gender		
Boys	38 (66.7)	
Girls	19 (33.3)	
Times of operation		
1	39 (68.4)	
2	11 (19.3)	
≧3	7 (12.3)	
Eye for operation		
Left	14 (24.6)	
Right	13 (22.8)	
Both	30 (52.6)	
Duration of disease		
<1 month	19 (33.3)	
1–3 months	14 (24.6)	
3–6 months	8 (14.0)	
6–12 months	4 (7.0)	
>12 months	12 (21.1)	

SD: standard deviation.

**Table 2:** Demographic data of the enrolled caregivers.

Variable	*n* (%)	Mean (SD)
Age (in years)		30.02 (4.85)
Gender		
Male	19 (33.3)	
Female	38 (66.7)	
Occupation status		
Employed	30 (52.6)	
Unemployed	27 (47.4)	
Education level		
Primary school or beneath	7 (12.3)	
Technical secondary school	9 (15.8)	
High school	20 (35.1)	
2-year college	7 (12.3)	
4-year university or above	14 (24.6)	
Household income per month		
<1000	2 (3.5)	
1001–3000	22 (38.6)	
3001–5000	15 (26.3)	
5001–7000	7 (12.3)	
>7000	11 (19.3)	

SD: standard deviation.

**Table 3:** The average magnitudes of caregiver's burden among the subjects.

Burden	Scale	Average (mean ± SD)	Range
Time-dependence	0–20	12.718 ± 5.53	0–20
Physical	0–16	7.53 ± 3.88	0–16
Development	0–20	9.34 ± 5.13	0–20
Social	0–20	5.85 ± 4.88	0–20
Emotional	0–20	3.02 ± 3.91	0–16
Total	0–96	38.44 ± 18.04	8–89

SD: standard deviation.

**Table 4:** The average magnitudes of positive aspects of caregiving among the subjects.

Positive aspects	Scale	Average (mean ± SD)	Range
Self-affirmation	0–30	16.88 ± 6.22	4–30
Outlook on life	0–15	9.24 ± 3.50	0–15
Total	0–45	26.12 ± 8.45	10–45

SD: standard deviation.

**Table 5:** Pearson correlation analysis of relationship between summary score of CBI and PAC.

		Aggregate score of PAC	Self-affirmation	Outlook on life
Aggregate score of CBI	Pearson correlation	−0.195	−0.131	−0.232
	Significance (two-sided test)	0.146	0.332	0.082

Physical	Pearson correlation	0.055	0.074	−0.001
	Significance (two-sided test)	0.686	0.582	0.995

Social	Spearman correlation coefficient	−0.243	−0.218	−0.226
	Significance (two-sided test)	0.068	0.103	0.092

Emotional	Spearman correlation coefficient	−0.327	−0.258	−0.382
	Significance (two-sided test)	0.013	0.053	0.003

Time-dependence	Spearman correlation coefficient	0.076	0.153	−0.108
	Significance (two-sided test)	0.572	0.257	0.425

Development	Pearson correlation	−0.23	−0.171	−0.244
	Significance (two-sided test)	0.085	0.202	0.068

**Table 6:** Generalized linear regression analysis of the summary score of the CBI measure as a function of demographic factors.

	*χ* ^2^	Degrees of freedom	*P*
Intercept	377.546	1	0
Duration of disease	10.922	4	0.027
Gender of caregiver	6.195	1	0.013
Occupation	8.379	1	0.004
Education	11.487	4	0.022
Household income	10.673	4	0.03

**Table 7:** Generalized linear regression analysis of the summary score of the developmental burden measure as a function of demographic factors.

	*χ* ^2^	Degrees of freedom	*P*
Intercept	200.594	1	0
Education	10.594	4	0.032

**Table 8:** Generalized linear regression analysis of the summary score of the physical burden measure as a function of demographic factors.

	*χ* ^2^	Degrees of freedom	*P*
Intercept	66.037	1	0
Gender of caregiver	3.985	1	0.046
Occupation	4.79	1	0.029

**Table 9:** Generalized linear regression analysis of the summary score of the time-dependence measure as a function of demographic factors.

	*χ* ^2^	Degrees of freedom	*P*
Intercept	0.08	1	0.778
Duration of disease	13.84	4	0.008
Age of patient	9.97	1	0.002
Age of caregiver	4.99	1	0.025
Household income	13.829	4	0.008

## Data Availability

The data used to support the findings of this study are available from the corresponding author upon request.
